# Outbreak of suspected pertussis in Kaltungo, Gombe State, Northern Nigeria, 2015: the role of sub-optimum routine immunization coverage

**DOI:** 10.11604/pamj.supp.2019.32.1.13352

**Published:** 2019-01-24

**Authors:** Ahmed Abubakar, Mahmud Dalhat, Abdulaziz Mohammed, Olayinka Stephen Ilesanmi, Uchenna Anebonam, Nyampa Barau, Sarafadeen Salami, Olawunmi Ajayi, Abba Shehu, Abisola Oladimeji, Saheed Gidado, Patrick Nguku, Ndadilnasiya Waziri, David Karatu, Peter Nsubuga

**Affiliations:** 1 Nigeria Field Epidemiology and Laboratory Training Program, Abuja, Nigeria; 2 Gombe State Ministry of Health, Nigeria; 3 Global Public Health Solutions, Atlanta GA, USA

**Keywords:** Pertussis, Kaltungo, Nigeria, outbreak, immunization

## Abstract

**Introduction:**

Despite the availability of vaccines, pertussis outbreaks still occur in developing countries. In December 2015 we investigated a pertussis outbreak in Kaltungo, Nigeria to identify determinants of infection and institute control measures.

**Methods:**

We enrolled 155 cases and 310 unmatched controls. We defined cases as residents of Kaltungo with paroxysmal or whooping cough lasting 2 weeks with or without vomiting and randomly selected neighborhood controls. Using structured questionnaire, we collected data on socio-demographics, clinical and risk factors. We collected twelve nasopharyngeal swabs for laboratory analysis using Polymerase Chain Reaction.

**Results:**

Median age was 24 months (range 1-132 months) for cases and 27 months (range 1-189 months) for controls. Female cases and controls were 86 (55.5%) and 150 (48.4%) respectively. A total of 83 (56.6%) cases were in age group 12-59 months. Age-specific-attack-rate was 83/1,786 (4.7%); Age-specific-case-fatality-rate was 21/83 (25.3%); Age-specific-proportional-mortality-ratio was 21/24 (87.5%). A total of 61 (39.4%) zero doses and 30.1% Pentavalent dropouts were documented. Multivariate analysis revealed parental refusal (adjusted OR = 27.8; CI = 8.8-87.7), contact with a case (AOR = 7.9, CI = 4.3-14.7, P = 0.000), belonging to the Muslim faith (AOR = 2.0; CI = 1.1-3.5) and having mothers with informal education only (AOR = 4.7, CI-2.6-8.4) as independent predictors of pertussis infection.

**Conclusion:**

Sub-optimal vaccination due to parental refusal and informal education of mothers were major determinants of pertussis infection. We conducted awareness campaigns of key immunization messages targeted at the informal education sector. We ensured appropriate case management, contact vaccination and health education in public gatherings, worship places and schools.

## Introduction

Pertussis, also known as whooping cough, is a highly communicable acute respiratory tract disease predominantly affecting children and caused by a gram-negative bacterial species, *Bordetella pertussis* [1]. The disease varies clinically from severe illness with frequent coughing paroxysms followed by an inspiratory whoop to very mild cases that may be mistaken for a cold with an incubation period of 7-10 days [2]. The World Health Organization (WHO) estimates that there are 60 million cases of pertussis with half a million to one million deaths per annum [2]. While good surveillance data reveals the re-emergence of pertussis in developed countries, pertussis surveillance data is largely missing for developing countries [3]. In 2008, pertussis caused an estimated 195,000 deaths worldwide [4]. Ninety percent of these outbreaks occur in developing countries [5]. The global Diphtheria, Pertussis, Tetanus (DPT) vaccination coverage moved from 5% in 1974 to 83% in 2011, yet, almost one-fifth of the world’s children had not received the DPT series during their first year of life [6]. Most of these unvaccinated children live in developing countries particularly Nigeria with a projected population of 182 million people in 2015 [7]. Epidemiological data from high-income countries show that, despite high vaccine coverage, the pertussis burden has increased in non-immunized or partially immunized infants [8]. This resurgence of pertussis has most likely arisen through a combination of factors: improved diagnostics, pathogen adaptation which may have reduced the efficacy of pertussis vaccines, waning immunity occurring after vaccination, vaccination which induces short duration of protection compared with natural infection with B. pertussis [9-12].

In Nigeria, the Expanded Programme on Immunization (EPI) recommends doses of pentavalent vaccine at ages 6, 10 and 14 weeks [6]. Pentavalent vaccine is a combination of five vaccines-in-one that prevents diphtheria, tetanus, whooping cough, hepatitis B and haemophilus influenza type B. Although polio vaccination is successful in Nigeria with over 95% coverage, it has not translated into a higher routine immunization with a national Pentavalent vaccination coverage of 70% as at 2014 [6, 10]. High immunization coverage with an effective vaccine is the mainstay of prevention and control of pertussis [11]. The availability of an effective vaccine against B. pertussis since the 1940s led to a substantial reduction in the morbidity and mortality caused by pertussis [12]. The occurrence of pertussis outbreaks reflects inadequate coverage of national childhood immunization programmes [13]. The recommended routine immunization and booster doses of vaccines should be considered for older children whose immunity may begin to wane [14]. Vaccination coverage in the northern parts of Nigeria at 56% is lower than the rest of the country at 70% [15]. An important reason for this low coverage in Gombe State is a refusal to participate in routine immunization due to the resistance of internally displaced persons that influx from neighbouring States, such as Adamawa, Yobe, and Borno, into Gombe State as a result of the insurgency, to participate in immunization programmes [16]. On the 5th December 2015, we noticed several children coughing, gasping for air with occasional shortness of breath and post-tussive vomiting in Kaltungo Local government area of Gombe state Nigeria. The children showed all the signs suggestive of pertussis and we investigated a suspected pertussis outbreak. Our objectives were to investigate the outbreak to confirm the existence of the outbreak, characterize the outbreak in person, place and time, identify associated risks and determinants of infection and to institute control measures.

## Methods

**Study setting:** Gombe State, formed out of the old Bauchi State is located in north-eastern Nigeria. The State has an area of 20,265 square kilometers and a population of about 2.3 million as at 2006 census [17]. Gombe State has 11 local government areas (LGA) and shares boundaries with Yobe State to the North, Adamawa and Taraba States to the South, Borno State to the East and Bauchi State to the West. Kaltungo is one of the Local Government Areas in Gombe State with the headquarters in Kaltungo town. Kaltungo has an area of 881 square kilometer, 10 wards or districts and a population of 149,805 as at the 2006 census [17]. Awak is a district in Kaltungo.

**Study design:** we conducted a 1:2 case-control study. We moved to the middle of the town near the borehole, and rolled a bottle, selection of cases commenced from where the bottle pointed to and systematically selected two controls randomly for every case selected. A relative and a neighbor were selected as controls. A total of 155 cases and 310 controls were recruited.

**Study population:** our study population was residents of Awak in Kaltungo Local Government area of Gombe State residing there for at least two weeks.

**Advocacy:** we started the investigation at Awak health centre on the 5th of December 2016. We paid advocacy visit to the community head and the in charge of the health centre. We checked the health registers and line listed the cases. We conducted active case search and traced the line listed cases to their home addresses.

**Case definition:** *suspected case*: any person having a cough illness lasting more than 2 weeks with any of these three symptoms: paroxysm, whooping sound, post-tussive vomiting with or without cyanosis. Control: a control was defined as any person not having any cough illness lasting more than 2 weeks.

**Study instruments:** we designed a structured questionnaire to collect and measure data on socio-economic status, demographic characteristics, clinical information, immunization history and risk factors.

**Data collection and procedure:** we recruited three health care workers in the Awak health post as research assistants and trained them on the use of the study instrument and conducting face-to-face interviews. Five residents of the Nigeria Field Epidemiology and Laboratory Training Program in addition to the trained research assistants collected data from the field for 5 days.

**Data management and analysis:** the data was entered into Epi Info 7.1 (US Centers for Disease Control and Prevention), cleaned and checked for inconsistencies before analysis. We summarized our findings using frequencies, means (with standard deviation) and proportion for univariate analysis. We checked for associations among all the independent variables against the dependent study outcome variable of interest at 95% Confidence Interval. All the significant variables were plugged into an unconditional logistic regression model to check for independent predictors’ of pertussis infections.

**Laboratory:** we collected 12 nasopharyngeal swabs using charcoal transport media and sent to the reference laboratory in Lagos. The laboratory uses a single-point indirect enzyme-linked immunosorbent assay (ELISA) for detection of immunoglobulin G (IgG) anti-bodies against pertussis; specimens with anti-pertussis IgG concentrations of ≥ 94 IU/mL were considered to be seropositive.

**Ethical consideration:** the State ministry of health was informed of our response activities. We obtained verbal consent from the parents or guardian of the children. Permission to review the line list of cases was obtained from the health facility. Data obtained was kept on a password protected computer and used only for the purpose of the outbreak response. The cases were all treated with erythromycin.

## Results

Out of 465 cases and control, age group 12-59 months was the most affected. Two hundred and thirty-six (51%) participants were female. Sixteen had no form of education while 2.4% had tertiary education, 58.1% belong to the Islamic faith. Seventy-two of the mothers were farmers while 1.7% were civil servants (Table 1). We enrolled 465 participants. A total of 155 cases and 310 controls were recruited with an overall attack rate of 155/11,172 (1.4%) and case fatality rate of 24/155 (15.5%). The most affected age group was 12-59 months (53.5%) with an age-specific-attack-rate of 83/1,786 (4.7%), age-specific-case-fatality-rate of 21/83 (25.3%) and age-specific-proportional-mortality-ratio was 21/24 (87.5%). The median age of participants was 26 months (range = 1-189 months). The median age for case was 24 months (range = 1-132 months) and 27 months (range = 1-189 months).

**Table 1:** Socio-demographic characteristics of pertussis cases and controls in Kaltungo, Gombe State, December 2015

Characteristics	Cases n=155		Controls n=310	
	N	%	N	%
**Age**				
1-11	32	20.7	84	27.1
12-59	83	53.6	12	3.9
60-119	35	22.6	144	46.5
120-179	5	3.2	3	1
180-190	0	0	67	21.6
**Sex**				
Female	86	55.5	150	48.4
Male	69	44.5	160	51.6
**Religion**				
Christianity	34	21.9	161	51.9
Islam	121	78.1	149	48.1
**Education**				
None	42	27.1	35	11.3
Primary	58	37.4	118	38.1
Quranic	27	17.4	5	1.6
Secondary	25	16.1	144	46.5
Tertiary	3	1.9	8	2.6
**Mother’s Occupation**				
Artisan	4	2.6	3	1
Civil servant	3	1.9	5	1.6
Farmer	114	73.6	218	71
Housewife	18	11.6	43	14
Others	0	0	2	0.7
Trader	16	10.3	36	11.7
Artisan	4	2.6	3	1

All of the cases in this outbreak had paroxysmal coughing and inspiratory whooping cough while 35% had post-tussive vomiting, 14.8% had apnea, 2% had cyanosis, 1.3% had epistaxis, 5.2% had hemoptysis, 1.9% had otitis media, 1.3% had pneumonia and 2.6% had seizures. Sixty percent of cases had the first dose of the pentavalent vaccine while 41.9% had Pentavalent 3, with a dropout rate of 30.1%. 49.4% of cases had zero doses, 17.4% had incomplete doses while only 43.2% had all three required doses of Pentavalent. 65% of cases waited for 3-7days before seeking medical attention. Eighty-five percent had contact with a case, 71.6% had received other immunizations. None of the cases and control had received a booster dose (Table 2). Contact with a case (OR = 8.8, 95% CI = 5.4 -14.3), mothers with only informal education (OR = 5.4, 95% CI = 3.4 -8.6) and parental refusal (OR = 20.2, 95% CI = 10.2 -39.8) were significantly associated with being infected with pertussis. Receiving Pentavalent 3 dose (OR = 0.3, 95% CI = 0.2 -0.4) and receiving other immunizations (OR = 0.3, 95% CI = 0.2 -0.5) were protective. Cases were also more likely to belong to the Muslim faith (OR = 3.9, 95% CI = 2.5 -6.0) (Table 3).

**Table 2:** Pentavalent history of pertussis cases and controls in Kaltungo, Gombe State, December 2015

Symptoms	Case N (%) n = 155	Controls N (%) n = 310
Zero doses	61 (39.4)	42 (13.6)
Pentavalent 1	93 (60.0)	261 (84.2)
Pentavalent 3	65 (41.9)	222 (71.6)
Drop out	30.1%	14.9%
Booster dose	0	0

**Table 3:** Factors associated with pertussis infection in Kaltungo, December 2015

Variable	Cases n = 155 N (%)	Controls n = 310 N (%)	OR	95% CI
Islam	121 (78.1)	149 (48.1)	3.9	2.5 - 6.0
Received other RI	111 (71.6)	275 (88.7)	0.3	0.2 - 0.5
Sub-Optimal dose	88 (56.8)	95 (30.7)	3	2.0 - 4.4
Received 3doses of Pentavalent	66 (42.6)	222 (71.6)	0.3	0.2 - 0.4
Heard of Whooping Cough	47 (30.3)	119 (38.4)	0.7	0.5 - 1.1
Parental refusal	66(42.6)	11 (3.6)	20.2	10.2 - 39.8
Informal Educated Mothers	69 (44.5)	40 (12.9)	5.4	3.4 - 8.6
Contact with a case	131 (84.5)	119 (38.4)	8.8	5.4 - 14.3

In our multivariate analysis, children who had parental refusal were 27.8 times more likely to have pertussis infection as compared with children whose parents accepted (AOR = 27.8, 95% CI = 8.8 -87.7). Also, children who had contact with a pertussis case were 7.9 times more likely to have pertussis infection than children who did not have contact with pertussis case (AOR = 7.9, 95% CI = 4.3 -14.7). Children whose mothers had informal education were 4.7 times more likely to have pertussis infection (AOR = 4.7, 95% CI = 2.6 -8.4) as compared with children whose mothers had formal education. Those who belong to the Muslim faith were two times more likely to have pertussis infection (AOR = 2.0, 95% CI = 1.1 -3.5) as compared with those of other faiths. There was no statistically significant difference on the knowledge of whooping cough between cases and controls and receiving other routine immunization vaccines was not significant at multivariate level (Table 4).

**Table 4:** Independent determinants of pertussis infection in Kaltungo, December 2015

Variable	AdjustedOdds Ratio	95% ConfidenceInterval	P-value
Islam	2.0	1.1 - 3.5	0.02
Received other RI	2.9	1.0 - 8.8	0.06
Sub-optimal dose	0.12	0.02 - 0.8	0.03
Received 3 doses of Pentavalent	0.07	0.01 - 0.5	0.007
Parental refusal	27.8	8.8 - 87.7	< 0.001
Informal educated mothers	4.7	2.6 - 8.4	< 0.001
Contact with case	7.9	4.3 - 14.7	< 0.001

## Discussion

This study aimed to investigate suspected pertussis outbreak in Kaltungo. The most affected age group in this study was 12-59 months, those less than 12 months were less affected. Older children were mostly affected due to a lack of booster vaccination in the study area. As reported in a study by Preziosi et al. in Senegal children < 5 years old represented 60 percent of the cases in 1986 [5]. Similar to our finding though among children less than 5 years those less than 12 months were less affected. Females were found to have a higher incidence of pertussis in this study. A similar observation was made by Preziosi et al. in Senegal, 1986 who reported that annual incidences were always higher among girls, but contrary to the report of Michel who reported no gender predilection in the occurrence of pertussis [5, 17]. The most frequently occurring symptoms were paroxysmal coughing, whooping cough and post-tussive vomiting which are the signs of pertussis. Several workers have reported similar clinical manifestations of pertussis [18, 19].

Vaccine coverage among cases was 50% with a dropout rate of 30.1%. The expected coverage of pentavalent vaccine is 85%. This huge gap shows that some children are unvaccinated against whooping cough thereby increasing their susceptibility to pertussis infection. Regarding completeness of all vaccines, 17.4% had incomplete vaccination while 43.2% had received complete doses of pentavalent vaccines. In 2010, national Pentavalent 3 coverage rates reached 69%. Low coverage of pentavalent vaccine in this study was attributed to the context of ongoing vaccine stock-outs and significant coverage heterogeneity among states [20]. Lack of adequate knowledge on causes, transmission and prevention of pertussis could have made it difficult for parents to prevent healthy children from contacting sick children thus resulting in the continuous spread of the infection. Parental refusal, having contact with a case and mothers having only informal education were the only independent predictors of pertussis infection which could also be due to lack of adequate knowledge on causes transmission and prevention of pertussis.

The high occurrence of cases among Muslims could be attributed to boycotts of immunization programmes by Muslim communities particularly in northern Nigeria largely due to claims that the vaccine could be contaminated with anti-fertility agents (estradiol hormone), HIV and cancerous agents [21, 22]. We had some limitations during our investigation. Firstly, unavailability of azithromycin to manage cases adequately led to the use erythromycin another drug of choice. Secondly, we could not collect samples immediately due to late arrival of transport media. Secondly, we lacked the diagnostic capacity to process samples in the nearby facility thereby leading to delay in effective response.

## Conclusion

Pertussis outbreak in this community occurred as a result of refusal to immunize the children. Sub-optimal immunization played a significant role in the propagation of this outbreak which is partly due to parental refusal, religious sentiments and low educational status of the mothers which are predictors of pertussis infection. We strengthened clinical treatment of cases with erythromycin and paracetamol. We continued active case search for more cases and also active surveillance. We enlightened the community on key immunization messages at the places of worships, schools and markets. We also targeted informal education sector for maximum impact. We encouraged the Disease Surveillance and Notification Officer and the State epidemiologist to be more vigilant to enable us to discover cases earlier.

**Recommendation:** campaign on key immunization messages should be sustained.

### What is known about this topic

- Despite the availability of vaccines, pertussis outbreaks still occur in developing countries;
- Without high index of suspicion pertussis can be mistaken for common cold.

### What this study adds

- Sub-optimal immunization plays significant role in the propagation of pertussis;
- With surveillance, active case search and prompt clinical management of cases outbreak of pertussis can be controlled.

## Competing interests

The authors declare no competing interests.
